# Characterization of Aroma Compounds in Cooked Sorghum Using Comprehensive Two-Dimensional Gas Chromatography-Time-of-Flight Mass Spectrometry and Gas Chromatography-Olfactometry-Mass Spectrometry

**DOI:** 10.3390/molecules26164796

**Published:** 2021-08-07

**Authors:** Shuang Chen, Li Wang, Derang Ni, Lin Lin, Heyu Wang, Yan Xu

**Affiliations:** 1 Key Laboratory of Industrial Biotechnology of Ministry of Education, State Key Laboratory of Food Science & Technology, School of Biotechnology, Jiangnan University, Wuxi 214122, China; yxu@jiangnan.edu.cn; 2 Technique Center of Kweichow Moutai Co. Ltd., Guizhou 564500, China; zmx1019@163.com (L.W.); derangni@163.com (D.N.); eileenjn@126.com (L.L.); moutai2@163.com (H.W.)

**Keywords:** cooked sorghum, aroma compounds, SPME, GC×GC-TOFMS, GC-O/MS

## Abstract

Sorghum is the major raw material for the production of Chinese Baijiu (Chinese liquor) and has a great effect on the flavor of Baijiu. Volatiles in cooked glutinous and non-glutinous sorghum samples were extracted using solid-phase microextraction (SPME) and analyzed via comprehensive two-dimensional gas chromatography-time-of-flight mass spectrometry (GC×GC-TOFMS) and gas chromatography-olfactometry/mass spectrometry (GC-O/MS). A total of 145 volatile compounds and 52 potent odorant compounds were identified from both sorghum types according to the retention index, MS, aroma, and standards. Based on their aroma features, the compounds were grouped into eight general categories, and the intensities of each aroma group were summed. Moreover, most of the compounds detected in the cooked sorghums were also detected in commercial Chinese Baijiu, indicating that the aroma compounds produced during the sorghum cooking process have a direct and significant influence on the final flavor quality of Baijiu.

## 1. Introduction

Sorghum one of the oldest crops, has been widely grown in the semiarid regions of the world [1]. It is the fifth most important cereal crop in the world and has been widely used in feed and food industries globally. However, sorghum is the most commonly used raw material for the production of Chinese Baijiu (Chinese liquor) [2]. Chinese Baijiu, also known as sorghum spirit, is a traditional Chinese distilled spirit and the most consumed spirit in the world (over 7 billion liters annually) [3]. Baijiu is usually distilled from fermented sorghum, and the demand for sorghum exceeds 4 million tons per year [3]. The production of Baijiu involves a typical repeated-batch fermentation and distillation process [4]. Fermented sorghum is usually mixed with soaked fresh sorghum to undergo solid-state distillation. Alcohol distillation and cooking of the fresh sorghum occur simultaneously. The aroma compounds generated during the cooking process are directly infused into the freshly produced Baijiu [3]. Therefore, the aroma compounds of cooked sorghum can greatly impact the final flavor quality of the Baijiu [5]. Depending on its amylose content, sorghum can be classified as non-glutinous or glutinous [5]. Both types of sorghums are widely used for Baijiu production, with the overall flavor characteristics of the Baijiu produced being significantly different [3].

As a pleasurable alcoholic beverage, the quality of Baijiu is highly dependent on its aroma quality, with the presence of volatile compounds being vital to its aroma characteristics. As far as Baijiu manufacturing is concerned, a significant amount of research on the aroma of Baijiu has already been carried out [6,7]. More than 300 aroma-active compounds have been identified in Baijiu, comprising numerous chemical classes, including esters, alcohols, fatty acids, pyrazines, and terpenes [8]. These aroma-active compounds may be derived from the raw materials, fermentation, distillation and aging processes used during production [9]. Since sorghum is the major raw material in the production of Chinese Baijiu, a few authors have identified the volatile compounds of sorghum. Using simultaneous distillation-extraction (SDE) and headspace-solid-phase microextraction (SPME) with gas chromatography-mass spectrometry (GC-MS), Lian et al. [10] identified 108 volatile compounds in sorghum.

However, not all of these compounds are aroma-active compounds. Gas chromatography-olfactometry (GC-O) is the most widely used analytical method for evaluating which volatile compounds are also important odorants in foods. Wu et al. [11] were the first to study the aroma-active compounds of cooked sorghum by GC-O/MS, tentatively identifying a total of 26 aroma-active compounds through comparison with the NIST retention index database. However, the aroma-active compounds that are responsible for the aroma profiles of different types of sorghum have not yet been clearly elucidated.

Therefore, the aims of this work were to: (I) develop an in-vial cooking method to capture and enrich the volatile compounds of cooked sorghum; (II) characterize the overall volatile compound profile by means of GC×GC-TOFMS; (III) characterize the aroma-active compounds of cooked sorghum by GC-O/MS; and (IV) compare and identify differences in the aroma profiles of glutinous and non-glutinous sorghum. The results of this study could provide a foundation for understanding the aroma role of sorghum as an ingredient in foods and beverages, such as Baijiu.

## 2. Results and Discussion

### 2.1. Optimization of the Aroma Extraction Method for Cooked Sorghum

**Figure 1:**
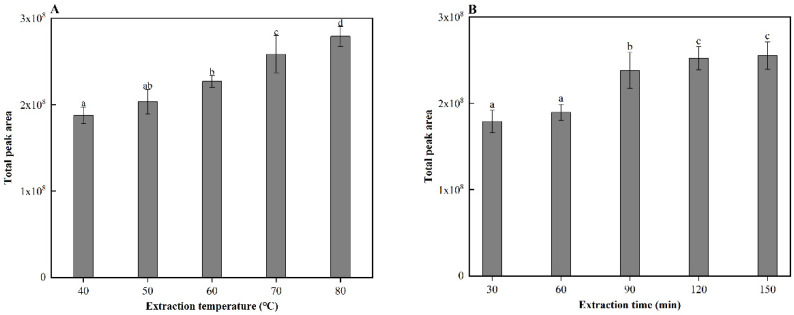
(**A**) extraction temperatures, and (**B**) extraction times of solid-phase microextraction (SPME) with corresponding gas chromatography-mass spectrometry (GC-MS) peak areas. Different letters indicate significant differences in values (*p* < 0.5).

Solid-phase microextraction (SPME) is widely used for the analysis of aroma compounds due to its relatively simple sample preparation and fully automated operation [12]. In this study, SPME was used to analyze the aroma compounds in cooked sorghum. Before analysis, the SPME conditions were optimized. Firstly, four SPME fibers with different coatings, PDMS, PDMS/DVB, CAR/PDMS, and DVB/CAR/PDMS were evaluated using GC-MS. Among them, the DVB/CAR/PDMS fiber, with a bipolar coating, had the highest extraction yield of aroma compounds. As a result, this fiber was selected to further optimize the extraction temperature and time of the SPME procedure. GC-MS analysis revealed that the volatile compound content of cooked sorghum increases significantly (*p* < 0.05) with increases in the extraction temperature (40–80 °C) (Figure 1A) and time (30–120 min) (Figure 1B). Therefore, 80 °C (upper temperature limit of the instrument) and 120 min were determined to be the optimal conditions for aroma extraction.

### 2.2. Analysis of Volatiles in Cooked Sorghum Samples by Using GC×GC-TOFMS

**Figure 2:**
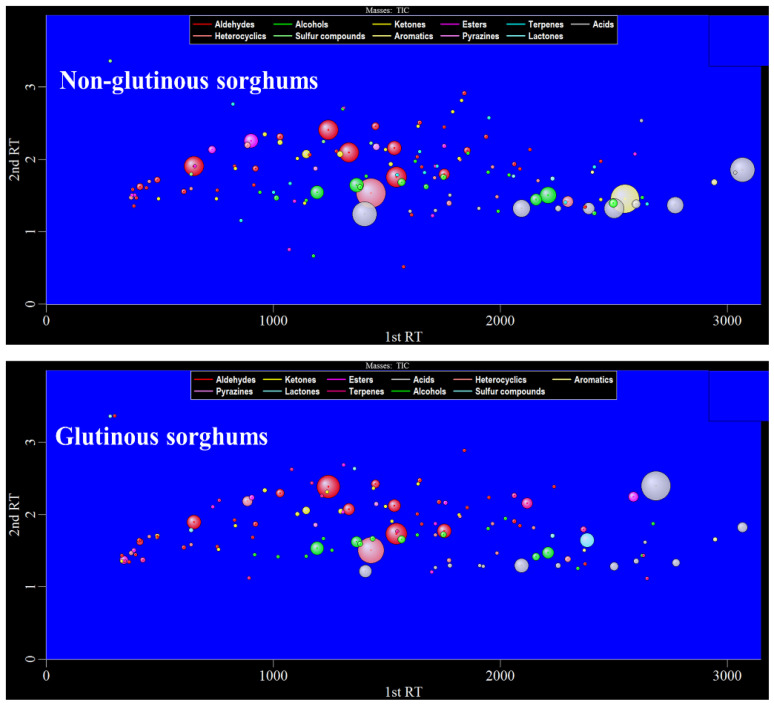
The 2D bubble plot of glutinous and non-glutinous sorghum samples.

Figure 2 shows the bubble plot obtained by GC×GC-TOFMS analysis of the volatile components of two cooked sorghum samples. Tentative compound identification was performed by firstly comparing database mass spectra with experimental ones, where database matches were considered with a spectral similarity of higher than 80%, and secondly, comparing between calculated and literature retention values, where a difference value within 50 is desirable. Thirdly, the positive identification of 98 compounds (~67% of the total number) was performed with commercially available pure standard compounds injected for qualitative analysis. Finally, a total of 145 volatile compounds were retained in this study, among which aldehydes were present in the highest number (34), followed by alcohol (25), ketones (19), esters (16), terpenes (14), acids (14), heterocyclics (12), sulfides (4), aromatics (3), pyrazines (2), and lactones (2) (Table 1).

**Figure 3:**
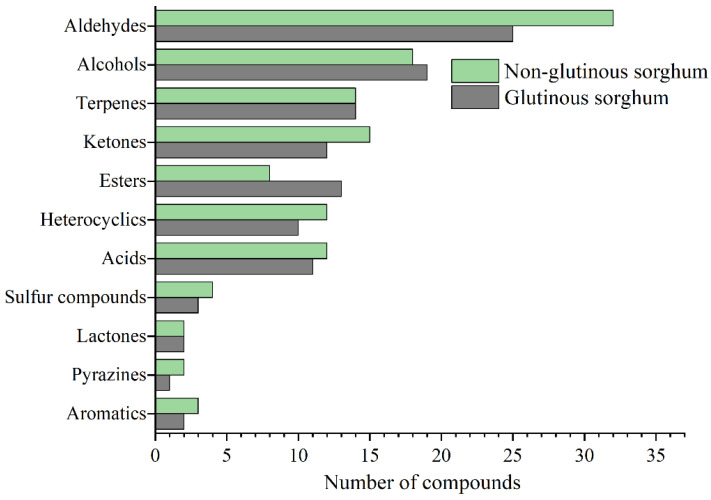
Comparison of identification compounds in cooked glutinous and non-glutinous sorghum obtained by HS-SPME-GC×GC-TOFMS.

As shown in Figure 3, aldehydes were the most abundant class of volatile compounds in the cooked sorghum samples and both saturated and unsaturated linear chain compounds were representative of this class [5]. Aldehydes usually derive from lipid oxidation, and these results could be related to the larger amount of lipids present in the bran layers. Saturated fatty aldehydes which are aromatics, have a greater contribution to the aroma of a cereal product because of their low odor threshold values (OAVs), and provide almond, malt, pungent (pentanal), grassy, green and fatty (hexanal), fatty, citrus and rancid (heptanal), fatty, soapy and green (octanal), fatty, citrus and green (nonanal) characteristics. (*E*)-2-enal is an important kind of aldehyde, there are (*E*)-2-heptenal, (*E*)-2-octenal, (*E*)-2-nonenal and (*E*)-2-decenal, respectively. Most of them show green, fruity, and fatty odors and the odor characters change with increases of the carbon chain length (Table 2). (*E*)-2-enals were not detected in the free aroma of original sorghum, but were detected in various aroma types of Baijiu, indicating that sorghum cooking can promote the introduction of fatty aldehydes into liquor, thus improving the flavor quality of baijiu.

Alcohols are usually produced by the decomposition of the secondary hydroperoxides of fatty acids. Pentanol, hexanol, heptanol and octanol are saturated fatty alcohols, mostly showing grass, flower and fruity odors. 1-octene-3-ol, also known as mushroom alcohol, has a typical mushroom smell, and the threshold in the air is only 1 μg/L [5].

Ketone compounds formed by the autoxidation of fatty acids were additional major volatile components in the cooked sorghum. Among the various ketones identified in the current study, most of them present butter, cream and fruity odors.

In total, 16 esters were identified in cooked sorghum, and they usually contributed to desirable odors, like fruity, floral and sweety.

Terpenes are a kind of natural hydrocarbon that are widely found in plants. In the current study a total of 14 terpenoid compounds were detected, including mono- and poly-terpene hydrocarbons, alcohols, carbonyls and esters.

A total of 14 acid compounds were identified in this study, of these identified acids, the homologous series of straight-chain monocarboxylic fatty acids from C4–C10, C14, and C16 were all detected, which contributed to acidic, rancid and cheesy odors.

Some heterocycles were also detected in the samples, including furan, furanone and pyrrole. 2-Pentylfuran exists in the cooking process of sorghum and it is derived from lipid peroxidation. This compound has a typical butter odor at a dilute concentration, but at higher concentrations, it has the less pleasant aroma characteristic of soybeans [13]. Furfural (sweet, woody and almond) which has been identified as an aroma-active compound in some cereals, was only detected in cooked samples.

### 2.3. Analysis of Aroma-Active Volatiles in Cooked Sorghums by Using GC-O/MS

**Figure 4:**
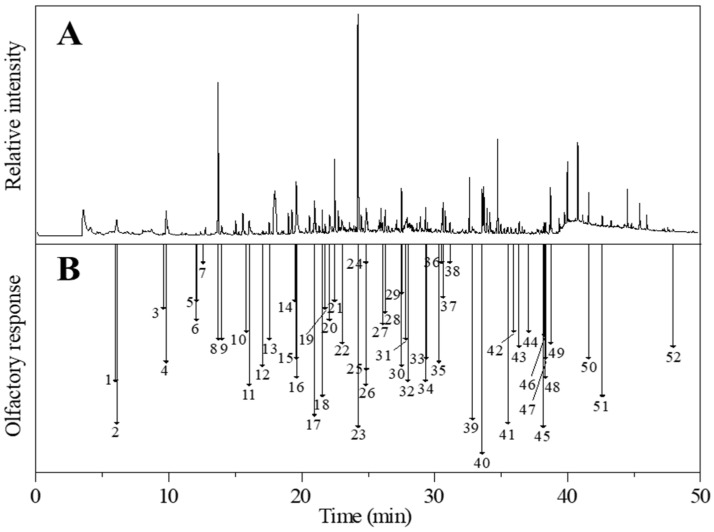
(**A**) Gas chromatography-mass spectrometry (GC-MS) and (**B**) gas chromatography-olfactory (GC-O) chromatograms of cooked glutinous sorghum. Numbers correspond to the compounds listed in Table 3.

The volatile compounds of the cooked glutinous and non-glutinous sorghum samples were directly extracted using the optimized headspace-SPME method and analyzed by GC-O/MS. The analysis results showed that 51 and 48 aroma-active volatiles were detected in glutinous and non-glutinous sorghum, respectively (Table 3). Most of the aroma compounds detected in the cooked sorghums have also previously been identified in Chinese Baijiu [6,7,14,15], indicating that the aroma compounds in sorghum contribute to the overall flavor of Baijiu. The 52 different identified aroma volatiles included 14 aldehydes, 13 terpenes, 8 acids, 5 ketones, 5 alcohols, 5 heterocyclic compounds, 1 sulfur-containing and 1 aromatic compound. Numerically, the aldehyde group comprised 27% of the total aroma-active volatiles in the cooked sorghum samples, followed by terpene (25%), acid (14%), ketone (10%), alcohol (10%), heterocyclic (10%), aromatic (2%), and sulphur-containing (2%) compounds. The aroma intensity of (*E*)-2-heptenal, nonanal, (*E*)-2-octenal, benzaldehyde, (*E*)-2-undecenal, (*E*, *E*)-2,4-decadienal, α-limonene, (-)-α-Cedrene, thujopsene, β-elemene, β-damascenone, geranyl acetone, 2,3-octanedione, acetophenone, 2-pentylfuran, 2-acetylpyrrole and dimethyl disulfide, present significant differences between the two samples (*p* < 0.05). (*E*)-2-undecenal was undetected in glutinous sorghum, and (*E*, *E*)-2,4-decadienal, α-limonene, thujopsene, and β-elemene were undetected in non-glutinous sorghum (Figure 4).

To estimate the contributions that these aroma-active volatiles had on the overall aroma impression, the 52 compounds were grouped into eight general aroma categories: sweet/fruity, green, floral, fatty, volatile acids, citrus-like, roasty/nutty, and miscellaneous (sulphury/smoky/mushroom). To compare the differences in the aroma intensities of each group between the cooked glutinous and non-glutinous sorghum samples, the intensities of each group were summed (Table 4).

#### 2.3.1. Sweet/Fruity

Sweet/fruity was found to be the major aroma character in both types of cooked sorghums, with the glutinous sorghum containing 12% more sweet/fruity aroma than the non-glutinous sorghum. Four types of compounds (terpenes, heterocyclics, alcohols, and ketones) provided the sweet/fruity aroma note in the cooked sorghums. Among the four terpenes (elemene, β-damascenone, geranyl acetone, and vanillin), β-damascenone and geranyl acetone were significant contributors to the overall aroma of the cooked sorghums. During the GC-O/MS analysis, although no obvious peak for β-damascenone was observed, an aroma of rich honey sweetness was sniffed at 26.84 min, with an RI of 1833 (Figure 4), and the compound was identified using its retention time and mass ions. β-Damascenone is an ionone isomer belonging to the isoprenoid class of compounds. These compounds often exude the aroma of lemon at low concentrations, while at high concentrations the aromas of apple, rose, and honey are expressed [16]. According to the literature, β-damascenone is an important aroma contributor to Chinese Baijiu [6], having extremely low aroma threshold values in air (0.002–0.004 ng/L) and water (0.00075–0.002 μg/L) [17]. β-Damascenone and geranyl acetone are both bound aroma compounds of sorghum, with geranyl acetone also being an important aroma compound in the soy sauce aroma type Baijiu [6]. Vanillin is another common aroma compound in Chinese Baijiu [18]. It has a sweet vanilla aroma feature with a low aroma threshold of 26 μg/L in chinese rice wine [17,19]. The two sweet/fruity heterocyclic compounds (γ-octalactone and γ-nonalactone) contributed a coconut aroma to the cooked sorghums. Both compounds are γ-lactones, a significant class of aroma lactones that mostly have coconut, sweet, and peach aromas [20]. γ-Octalactone and γ-nonalactone also have relatively low aroma thresholds, 7 and 30–65 μg/L, respectively. Additionally, two alcohols and two ketones contributed to the sweet/fruity aromas of the cooked sorghums. Among these, phenethyl alcohol was a significant aroma contributor having an aroma intensity of 3.50 and 4.17 in glutinous and non-glutinous sorghum, respectively (Table 3).

#### 2.3.2. Green

Green was another significant aroma note in the cooked sorghums. In this study, 11 green aroma compounds were detected, having total aroma intensities 20% higher in glutinous versus non-glutinous sorghum (Table 4). Seven of the eleven compounds were aldehydes. Saturated aldehydes and (*E*)-2-enal unsaturated aldehydes often demonstrate grass and malty aromas [14,17]. Nonanal and 3-methylbutanal (saturated aldehydes) and (*E*)-2-octenal (an unsaturated aldehyde), were evaluated as having strong aroma intensities in both cooked sorghums. Lipids in sorghum are susceptible to oxidization, and the heat imposed during sorghum distillation may accelerate the oxidation process [21], possibly contributing to the presence of aldehyde compounds in the cooked sorghum samples. (*E*)-2-Enal aldehydes have not been detected in uncooked sorghum [10,11], while they have been detected in a variety of Chinese Baijiu [14], thus further substantiating that the distillation (cooking) process of sorghum may improve the release of bound aroma compounds and these compounds influence the final flavor quality of Baijiu. (-)-α-Cedrene, a terpene, showed the strongest green aroma note (5.0) in glutinous sorghum (Table 3). As (-)-α-Cedrene is also an aroma compound in the soy sauce aroma type Baijiu, this compound may strongly contribute to the overall aroma of Baijiu [22].

#### 2.3.3. Floral

Six compounds were detected as having floral aromas in the cooked sorghums. The summed aroma intensities of these compounds for the two types of sorghum were similar (Table 4). Of the compounds primarily responsible for the floral aromas of the cooked sorghums, acetophenone had the highest relative floral aroma intensity, 4.00 and 3.08 in glutinous and non-glutinous sorghum, respectively.

#### 2.3.4. Fatty

Seven compounds were detected as having fatty aromas. Compounds with long carbon chains, such as octanal and decanoic acid, often demonstrate fatty aromas. However, with decreases in the carbon chain length, compounds may demonstrate different aroma notes, and their aroma thresholds may also decrease [17].

#### 2.3.5. Volatile Acids

Organic acids are important aroma compounds in many traditional fermented foods [23]. Six compounds having acids aromas were detected, and their summed aroma intensities were 14% lower in glutinous versus non-glutinous sorghum (Table 4). Among these, hexanoic acid was evaluated as having sweaty and cheese aromas, with aroma intensities of 5.00 for both types of cooked sorghums (Table 3).

#### 2.3.6. Citrus-Like

The citrus-like aroma is a unique feature of the aroma profile of cooked sorghums. Four terpenes and one ketone were detected with citrus-like aromas. Among these, γ-terpinene showed a relatively strong aroma intensity of 2.00 both in glutinous and non-glutinous sorghum (Table 3).

#### 2.3.7. Roasty/Nutty

Three compounds were detected as having roasty/nutty aromas. Benzaldehyde demonstrated strong almond aroma intensities of 5.00 and 4.33 in glutinous and non-glutinous sorghum, respectively (Table 3).

#### 2.3.8. Miscellaneous

Besides the typical aroma notes of cooked sorghum, 1-octen-3-ol (mushroom aroma), dimethyl disulfide (onion aroma), and 2-methoxy-4-vinylphenol (smoky aroma), were also detected and contributed to the overall aromas of the cooked sorghums (Table 3).

Based on the ANOVA, the summed intensity of four aroma categories: sweet/fruity, green, volatile acids and citrus-like, present significant differences between glutinous and non-glutinous sorghum samples (Table 4).

## 3. Materials and Methods

### 3.1. Reagents and Chemicals

Two sorghum cultivars (glutinous and non-glutinous) produced in Guizhou Province in 2018, were provided by a liquor producing enterprise. The aroma standards were purchased from Sigma Aldrich (Shanghai) Trade Co. Ltd. All compounds were of chromatographic purity, and the purity was >95%. The n-alkane (C7–C30) for the linear retention index (RI) determination was obtained from Sigma-Aldrich Co., Ltd. (Shanghai, China). Ultrapure water was obtained from a Milli-Q purification system (Millipore, Bedford, MA, USA).

### 3.2. Sorghum Cooking

Sorghum samples were first moistened by adding 300 g of sorghum to 400 mL of distilled water in a 1 L beaker. The mixture was then sealed with foil and heated in an oven at 75 °C overnight [24]. Then, 5 g of the moist sorghum and 1 mL of distilled water were added to a 20 mL headspace glass vial that was purged with nitrogen gas and loosely sealed with a Teflon-coated septum screw cap. Next, the vial was placed in a boiling water bath (100 °C) for 1 h, and directly subjected to headspace-solid-phase microextraction.

### 3.3. Optimization of Headspace-Solid-Phase Microextraction Sampling

Four SPME fiber types, polydimethylsiloxane (PDMS), PDMS/divinylbenzene (DVB), carboxen (CAR)/PDMS, and DVB/CAR/PDMS, were evaluated for their capabilities at extracting and concentrating the headspace volatiles of cooked sorghum. Extraction temperatures (40, 50, 60, 70, and 80 °C) and times (30, 60, 90, 120, and 150 min) were evaluated to determine the optimal conditions for extracting the volatiles using each type of SPME fiber. After optimization, the DVB/CAR/PDMS SPME fiber was inserted into the headspace vial of the sorghum sample and left for 120 min at 80 °C. Subsequently, the fiber was inserted into the injection port of the GC-MS for 5 min at 250 °C for thermal desorption of the flavor volatiles.

### 3.4. HS-SPME-GC×GC-TOFMS Instrumentation

Following a method presented in the literature [25], experiments were performed on a LECO Pegasus^®^ 4D GC×GC-TOFMS system (LECO Corp., St. Joseph, MI, USA). The column configurations for GC×GC analysis consisted of two columns which were coupled in tandem. The 1D column was a 60 m × 0.25 mm × 0.25 µm DB-FFAP (Agilent Technologies, Palo Alto, CA, USA), and the 2D column was a 1.5 m × 0.25 mm × 0.25 µm Rxi-17Sil MS secondary column (Restek, Bellefonte, PA, USA). Helium was used as the carrier gas at a constant flow rate of 1.0 mL/min. The injector temperature was set at 250 °C. The initial oven temperature was held at 45 °C for 3 min, and then ramped at a rate of 4 °C/min to 150 °C, and held for 2 min, reaching 200 °C at 6 °C/min and 230 °C at 10 °C/min for 20 min. The secondary oven was set at a 5 °C offset, relative to the primary oven. The modulator was set at a 20 °C offset, relative to the primary oven, and a modulation period of 4 s was used. The TOFMS was operated at an acquisition rate of 100 spectra/s and scanned from 50 m/z to 350 m/z.

The detector voltage was set to 1350 V, and the electron energy was −70 V.

### 3.5. Gas Chromatography-Olfactometry/Mass Spectrometry (GC-O/MS) Analysis

Gas chromatography was performed using an Agilent 6890N GC equipped with an Agilent 5975 mass selective detector (Agilent Technologies, Santa Clara, CA) and olfactometer (ODP 2, Gerstel GmbH and Co. KG, Mulheim, Germany). Samples were separated and evaluated using a polar DB-FFAP column (60 m × 0.25 mm i.d, 0.25 μm film thickness, Agilent Technologies, Inc., Santa Clara, CA). The oven temperature was programmed to 40 °C for 2 min, then increased at a rate of 4 °C/min to 230 °C, and maintained there for 10 min. The carrier gas used was helium, at a flow rate of 2.0 mL/min. A split ratio of 1:1 was used for injection.

Half of the carrier flow was diverted to the olfactory port, and mixed with warm, humid air for sniffing. The sniffing port temperature was set to 250 °C. Three trained panelists (two females and one male) received two months of training on GC-O involving, at a minimum, 30 aroma-active reference compounds at concentrations that were 10 times their odor thresholds in air. During GC operation, each panelist placed his/her nose near the olfactory port to record the retention times, intensity values, and aroma descriptions of the samples. Odor intensities were evaluated using a 6-point intensity scale from 0 to 5, with “0” = no, “3” = moderate, and “5” = extreme intensity. Each panelist conducted the GC-O evaluation four times. The final intensity value of each aroma was an average of the aroma intensities obtained from all of the panelists.

The other half of the carrier flow was diverted to the mass spectrometer. The ion source temperature was set to 230 °C, electron impact ionization was applied in positive ion mode (70 eV), and the mass range scanned was m/z = 25~300.

### 3.6. Identification of Aroma Compounds

Identifications were made through comparisons of the aromas, mass spectra, and linear retention indices (LRIs) with reference/standard compounds. Aroma determinations were made by comparing the odor descriptions of the compounds analyzed by GC-O to Flavornet and Flavor DB (citations). Mass determinations were made by comparing the mass spectra of the compounds to the NIST/EPA/NIH mass spectral library (NIST 14, version 2.0) (NIST, Gaithersburg, MD). LRI analyses involved comparisons of the aroma LRIs with those of reference compounds. The LRIs of the odorants were calculated from the linear retention times of n-alkanes (C5–C30) using a DB-FFAP column according to a modified Kovats method [26]. All of the aroma-active compounds detected by GC-O were also confirmed through the use of standard compounds.

### 3.7. Statistical Analysis

The analysis of variance (ANOVA) was performed using Microsoft Excel 2013 software. Significant differences were estimated as “***”, *p* < 0.001 (very highly significant); “**”, *p* < 0.01 (highly significant); “*”, *p* < 0.05 (significant); “ns”, *p* > 0.05 (not significant).

## 4. Conclusions

In this study, an SPME method for the extraction of aroma compounds from cooked sorghums was developed. Using this method, a total of 145 volatile compounds and 52 aroma-active compounds were detected in cooked glutinous and non-glutinous sorghum by GC×GC-TOFMS and GC-O/MS respectively. These compounds were further identified through comparisons with reference/standard compounds. The aroma features and intensities of these compounds were evaluated by three well-trained panelists using olfactory detection. The aroma intensity of (*E*)-2-heptenal, nonanal, (*E*)-2-octenal, benzaldehyde, (*E*)-2-undecenal, (*E*, *E*)-2,4-decadienal, α-limonene, (-)-α-Cedrene, thujopsene, β-elemene, β-damascenone, geranyl acetone, 2,3-octanedione, acetophenone, 2-pentylfuran, 2-acetylpyrrole and dimethyl disulfide, present significant differences between the two samples (*p* < 0.05). (*E*)-2-undecenal was undetected in glutinous sorghum, and (*E*, *E*)-2,4-decadienal, α-limonene, thujopsene, and β-elemene were undetected in non-glutinous sorghum. Based on their aroma features, the compounds were grouped into eight general aroma categories, and the aroma intensities of each aroma group were totaled. The summed intensity of four aroma categories: sweet/fruity, green, volatile acids and citrus-like, present significant differences between glutinous and non-glutinous sorghum samples (*p* < 0.05). Glutinous sorghum contained 12%, 20% and 21% more sweet/fruity, green and citrus-like total intensities, respectively than non-glutinous sorghum; and contained 14% less acid total intensity, emphasizing the aromatic differences between the two cooked sorghums. In addition, most of the compounds detected in the cooked sorghums were also detected in commercial Baijiu, indicating that the aroma compounds produced during sorghum distillation (cooking) have direct and significant influences on the final flavor quality of Baijiu.

## Figures and Tables

**Table 1:** Comparison of volatile compounds detected in cooked glutinous and non-glutinous sorghum by HS-SPME-GC×GC-TOFMS.

Class	Number of Compounds		
	Glutinous	Non-Glutinous	Total
Phenol	2	3	3
Pyrazines	1	2	2
Lactones	2	2	2
Sulfur compounds	3	4	4
Acids	11	12	14
Heterocyclics	10	12	12
Esters	13	8	16
Ketones	12	15	19
Terpenes	14	14	14
Alcohols	19	18	25
Aldehydes	25	32	34
Total	112	122	145

**Table 2:** Volatile compounds identified in cooked glutinous and non-glutinous sorghums by HS-SPME-GC×GC-TOFMS.

Compounds	Peak Area		RI		Aroma ^c^	Identification ^d^
	Glutinous Sorghum	Non-Glutinous Sorghum	Cla-RI ^a^	Lit-RI ^b^		
**Aldehydes**						
Acetaldehyde	9,675,368 ± 350,920	2,416,216 ± 104,048	709	718	pungent, ether, fresh	RI, MS, STD
Butanal	903,728 ± 59,123	22,033,094 ± 215,879	875	854	pungent, green	RI, MS, Tent
2-Propenal	95,002 ± 8637	164,626 ± 10,770	832	838	almond, cherry	RI, MS, Tent
2-Methyl-2-propenal	53,945 ± 4904	95,014 ± 6216	887	893	wild, hyacinth, foliage	RI, MS, STD
2-Methyl-butanal	989,169 ± 64,712	34,559,663 ± 61,488	904	910	malt	RI, MS, STD
3-Methyl-butanal	2,194,192 ± 79,582	2,973,227 ± 45,278	917	916	cocoa, almond	RI, MS, STD
Pentanal	923,054 ± 60,387	100,296,059 ± 69,876	985	976	almond, malt, pungent	RI, MS, Tent
2-Butenal	162,609 ± 6776	842,320 ± 113,039	1056	1042	floral	RI, MS, Tent
Hexanal	2,355,706 ± 85,440	6,868,006 ± 104,589	1095	1083	grass, tallow, fatty	RI, MS, STD
(*E*)-2-Pentenal	-	804,648 ± 655,501	1125	1147	strawberry, fruity	RI, MS, STD
Heptanal	105,864 ± 4411	475,576 ± 63,822	1189	1178	fatty, citrus, rancid	RI, MS, Tent
3-Methyl-2-butenal	89,259 ± 8115	101,051 ± 6611	1212	1215	sweet fruity	RI, MS, Tent
2-Hexenal	197,246 ± 8219	1,106,558 ± 14,849	1207	1216	apple, green	RI, MS, STD
Octanal	1,390,299 ± 50,426	2,519,793 ± 38,373	1310	1319	fatty, soapy, green	RI, MS, STD
(*E*)-2-Heptenal	590,306 ± 38,618	-	1333	1320	soapy, fatty, almond	RI, MS, STD
(*E*)-2-Octenal	3,337,651 ± 121,055	2,579,505 ± 39,282	1443	1436	green	RI, MS, STD
Nonanal	11,917,467 ± 432,240	15,915,159 ± 685,342	1403	1422	fatty, citrus, green	RI, MS, STD
2-Octenal	-	14,342,203 ± 116,837	1443	1445	fatty green herbal	RI, MS, Tent
Decanal	1,631,776 ± 59,184	2,340,543 ± 35,643	1506	1506	soapy, orange peel	RI, MS, STD
Nonenal	-	11,040,264 ± 89,938	1440	1453	green cucumber	RI, MS, STD
(*E*)-2-Nonenal	829,816 ± 54,287	-	1520	1535	cucumber, fatty, green	RI, MS, STD
Benzaldehyde	3,571,972 ± 129,554	15,600,161 ± 23,756	1549	1524	almond, burnt, sugar	RI, MS, STD
(*E*, *Z*)-2,6-Nonadienal	-	1,781,208 ± 14,510	1597	1591	cucumber, wax, green	RI, MS, STD
Undecanal	-	2,441,022 ± 19,885	1611	1609	oil, pungent, sweet	RI, MS, STD
2,4-Octadienal	-	832,551 ± 678,232	1575	1585	green, cucumber	RI, MS, STD
(*E*)-2-Decenal	-	1,776,196 ± 14,469	1650	1641	tallow, waxy, fatty	RI, MS, STD
Phenylacetaldehyde	16,153,454 ± 585,877	26,877,978 ± 11,574	1658	1650	honey	RI, MS, STD
Dodecanal	-	1,731,008 ± 141,015	1715	1709	soapy, citrus, green	RI, MS, Tent
(*E*, *E*)-2,4-Nonadienal	729,557 ± 47,728	48,460,352 ± 474,815	1713	1686	fatty, tropical fruity	RI, MS, STD
(*E*)-2-Undecenal	510,997 ± 3430	5,568,899 ± 56,396	1719	1736	soapy, fatty, green	RI, MS, STD
(*E*, *E*)-2,4-Decadienal	466,460 ± 30,516	40,596,386 ± 397,762	1778	1783	fried, wax, fatty	RI, MS, STD
2,4-Dimethyl-benzaldehyde	96,234 ± 4010	158,918 ± 21,327	1748	1710	cherry, almond, vanilla	RI, MS, Tent
2-Methyl-undecanal	384,132 ± 3806	357,289 ± 18,627	1609	1636	waxy, fatty, metallic	RI, MS, Tent
1H-Pyrrole-2-carboxaldehyde	-	179,875 ± 46,534	2046	2059	musty, beefy, coffee	RI, MS, Tent
**Alcohols**						
2-Propanol	-	2,823,662 ± 23,002	921	950	musty, woody	RI, MS, Tent
2-Methyl-butanol	-	284,448 ± 12,249	1200	1214	wine, onion	RI, MS, STD
Pentanol	4,122,806 ± 149,532	-	1261	1275	fruity	RI, MS, STD
3-methyl-3-butenol	87,123 ± 3630	111,599 ± 14,977	1136	1118	sweet fruity	RI, MS, STD
2-Heptanol	4,272,658 ± 54,967	-	1316	1280	mushroom	RI, MS, STD
(*Z*)-2-Pentenol	99,697 ± 4154	115,061 ± 15,441	1310	1327	green, metallic	RI, MS, STD
Hexanol	16,714,881 ± 60,239	5,390,684 ± 22,135	1356	1350	resin, flower, green	RI, MS, STD
3-Hexenol	-	621,386 ± 506,208	1393	1398	green leafy	RI, MS, Tent
1-Octen-3-ol	8,675,498 ± 314,656	6,647,311 ± 26,248	1442	1456	mushroom	RI, MS, STD
Heptanol	1,929,513 ± 69,983	431,169 ± 6566	1440	1421	green	RI, MS, STD
2-Ethyl-hexanol	-	983,534 ± 42,353	1490	1481	rose, green	RI, MS, Tent
(*E*)-2-Heptenol	133,566 ± 5565	-	1531	1546	pungent, fatty	RI, MS, Tent
2-Nonanol	980,889 ± 64,171	8,535,137 ± 862,710	1555	1532	cucumber	RI, MS, Tent
Octanol	3,985,051 ± 144,536	1,399,177 ± 21,308	1565	1572	moss, nut, mushroom	RI, MS, STD
(*Z*)-3-Octenol	45,855 ± 4169	-	1566	1563	melon, earthy	RI, MS, Tent
4-Methoxy-benzenemethanol	-	31,493 ± 1356	1161	1178	flower, cherry, vanilla	RI, MS, Tent
(*E*)-2-Octenol	723,534 ± 47,334	-	1616	1645	mushroom	RI, MS, Tent
4-Methyl-pentanol	75,541 ± 6868	-	1334	1316	nutty	RI, MS, Tent
Nonanol	1,639,336 ± 59,458	728,439 ± 11,093	1663	1651	fatty, green	RI, MS, STD
Decanol	194,979 ± 8124	267,183 ± 35,856	1761	1784	fatty	RI, MS, Tent
2-Undecanol	153,446 ± 6394	291,288 ± 39,091	1734	1717	fresh wax	RI, MS, Tent
1-Hepten-3-ol	-	299,299 ± 43,822	1346	1331	sweet, green	RI, MS, STD
Benzenemethanol	544,118 ± 35,597	102,365,753 ± 297,758	1885	1902	sweet, flower	RI, MS, STD
Phenylethyl alcohol	4,612,402 ± 167,289	6,865,240 ± 295,633	1900	1931	honey, spice, rose	RI, MS, STD
2-Phenoxy-ethanol	48,375 ± 4398	-	2126	2142	rose, cinnamyl	RI, MS, Tent
**Ketones**						
2-Propanone	-	493,389 ± 1936	791	816	solvent, pear	RI, MS, Tent
2-Pentanone	-	2,038,749 ± 16,608	963	1003	ether, fruity	RI, MS, Tent
2,3-Butanedione	940,134 ± 34,098	-	965	977	butter, sweet, creamy	RI, MS, STD
2,3-Pentanedione	99,279 ± 9026	-	1086	1062	sweet, butter, creamy	RI, MS, Tent
2-Heptanone	-	6,160,141 ± 18,317	1213	1191	soap	RI, MS, Tent
3-Octanone	-	4,162,680 ± 91,099	1226	1252	soap, gasoline	RI, MS, Tent
2-Octanone	2,021,178 ± 73,307	4,559,765 ± 69,438	1261	1309	herb, butter, milk	RI, MS, STD
3-Hydroxy-2-butanone	192,650 ± 8027	-	1269	1277	butter, cream	RI, MS, STD
6-Methyl-5-hepten-2-one	1,476,409 ± 53,549	3,481,086 ± 53,012	1315	1342	pepper, mushroom	RI, MS, Tent
2,3-Octanedione	4,153,568 ± 61,383	3,576,519 ± 5446	1325	1330	cream, sweet	RI, MS, STD
2-Nonanone	73,548 ± 6686	-	1390	1385	sweet, coconut	RI, MS, STD
3-Octen-2-one	1,637,944 ± 59,408	2,551,762 ± 38,860	1391	1408	nut, crushed bug	RI, MS, Tent
3-Penten-2-one	-	325,033 ± 264,786	1159	1120	fruity, phenolic	RI, MS, Tent
2-Decanone	-	2,296,398 ± 1,870,745	1466	1480	flower, fatty	RI, MS, Tent
3-Nonen-2-one	403,732 ± 26,413	33,365,624 ± 691,571	1493	1520	berry	RI, MS, Tent
3,5-Octadien-2-one	-	638,633 ± 52,025	1538	1563	green grassy	RI, MS, Tent
2-Undecanone	1,521,845 ± 55,197	603,365 ± 9189	1571	1592	orange, fresh, green	RI, MS, STD
Acetophenone	5,212,317 ± 189,048	3,985,709 ± 17,163	1667	1655	floral	RI, MS, STD
4-(5-Methyl-2-furanyl)-2-butanone	44,108 ± 4010	242,127 ± 15,840	1769	1745	bitter, roasted	RI, MS, STD
**Esters**						
Ethyl formate	2,930,691 ± 10,629	4,730,110 ± 72,032	806	842	pungent	RI, MS, STD
Acetic acid, ethyl ester	2,141,451 ± 77,669	2,424,633 ± 36,924	864	902	fruity, sweet	RI, MS, STD
3-Methylbutanoic acid, ethyl ester	339,055 ± 33,600	-	1078	1067	fruity	RI, MS, STD
Pentanoic acid, ethyl ester	-	1,340,571 ± 10,920	1112	1159	yeast, fruity	RI, MS, STD
Hexanoic acid, methyl ester	151,637 ± 6318	-	1189	1183	fruity, fresh, sweet	RI, MS, Tent
Hexanoic acid, ethyl ester	2,006,843 ± 72,787	1,214,227 ± 18,491	1199	1240	apple, sweet	RI, MS, STD
Acetic acid, hexyl ester	262,775 ± 26,041	-	1265	1277	herb, fruity, sweet	RI, MS, STD
2-Hydroxy- propanoic acid, ethyl ester	6,528,852 ± 23,678	7,666,361 ± 33,013	1321	1345	fruity	RI, MS, Tent
Trans-2-octenoic acid, ethyl ester	70,963 ± 6451	-	1534	1540	fruity, pear, plum	RI, MS, Tent
Octanoic acid, ethyl ester	115,957 ± 4832	-	1413	1435	fruity, wine	RI, MS, STD
Heptanoic acid, methyl ester	101,821 ± 4243	-	1283	1292	sweet fruity, green	RI, MS, Tent
Hexanoic acid, pentyl ester	21,086 ± 1917	-	1513	1501	green fruity	RI, MS, STD
Hexanoic acid, hexyl ester	87,149 ± 7923	-	1585	1599	apple peel, peach	RI, MS, STD
Benzoic acid, methyl ester	-	1,546,633 ± 12,599	1612	1637	prune, lettuce, herb	RI, MS, Tent
Benzoic acid, 1-methylethyl ester	-	8,085,255 ± 65,659	1637	1639	sweet, fruity, floral	RI, MS, Tent
Hexadecanoic acid, ethyl ester	635,304 ± 41,562	161,126,361 ± 157,871	2228	2240	waxy, fruity	RI, MS, Tent
**Terpenes**						
α-Terpinene	383,922 ± 38,047	276,282 ± 14,404	1162	1172	citrus-like, floral	RI, MS, STD
α-Limonene	273,432 ± 27,097	161,047 ± 8396	1193	1200	citrus-like	RI, MS, STD
γ-Terpinene	116,456 ± 4853	172,494 ± 2314	1230	1238	citrus-like, lemon	RI, MS, STD
Terpinolene	154,506 ± 6438	240,321 ± 3225	1306	1280	citrus-like, floral	RI, MS, STD
β-Elemene	168,909 ± 7038	343,075 ± 46,041	1598	1586	fruity, sweet	RI, MS, STD
(-)-α-Cedrene	180,884 ± 7537	367,397 ± 49,305	1568	1571	cypress	RI, MS, STD
Thujopsene	255,330 ± 25,303	150,385 ± 7840	1600	1606	cypress	RI, MS, STD
β-Terpineol	108,746 ± 4531	161,074 ± 21,616	1632	1616	clove	RI, MS, STD
Linalool	80,301 ± 7300	130,720 ± 8552	1552	1535	flower, wood	RI, MS, STD
β-Damascenone	402,071 ± 3984	366,180 ± 19,090	1832	1827	sweet, honey	RI, MS, STD
Geranyl acetone	578,093 ± 37,819	14,052,357 ± 37,684	1861	1862	fruity	RI, MS, STD
(*E*)-β-ionone	334,172 ± 33,116	267,967 ± 13,970	1952	1953	violet	RI, MS, STD
D-nerolidol	343,040 ± 33,995	231,477 ± 12,068	2272	2290	mild floral	RI, MS, STD
(*E*)-Nerolidol	299,822 ± 29,712	387,590 ± 20,206	2040	2054	rose, apple	RI, MS, STD
**Acids**						
Acetic acid	18,084,293 ± 655,907	23,545,032 ± 1,013,901	1415	1398	acid, sour	RI, MS, STD
2-Methyl-propanoic acid	90,203 ± 3759	-	1565	1524	rancid, butter, cheese	RI, MS, STD
Butanoic acid	805,495 ± 8566	507,462 ± 6012	1625	1628	acid, sweaty	RI, MS, STD
3-Methyl-butanoic acid	-	593,181 ± 25,544	1660	1648	sweat, acid, rancid	RI, MS, STD
Pentanoic acid	656,331 ± 42,938	-	1737	1733	acidic, cheesy	RI, MS, STD
Hexanoic acid	32,242,500 ± 1,169,417	15,491,525 ± 667,099	1850	1857	fatty, sweat, cheese	RI, MS, STD
Heptanoic acid	8,631,813 ± 313,071	1,850,130 ± 79,671	2134	2142	rancid, sour, cheesy	RI, MS, STD
Octanoic acid	11,893,186 ± 431,359	3,281,620 ± 141,314	1472	1462	sweat, cheese	RI, MS, STD
Nonanoic acid	11,290,020 ± 409,483	3,026,817 ± 130,342	2164	2180	green, fatty	RI, MS, STD
Decanoic acid	1,549,490 ± 56,199	1,115,042 ± 16,981	2253	2264	rancid, fatty	RI, MS, STD
Hydrocinnamic acid	-	27,120 ± 1168	2603	2633	sweet, fatty, rose	RI, MS, STD
Hexadecanoic acid	-	38,378,443 ± 165,266	2886	2876	waxy, creamy fatty	RI, MS, Tent
Benzoic acid	1,480,735 ± 53,706	3,555,504 ± 54,145	2410	2404	urine	RI, MS, STD
Tetradecanoic acid	2,554,084 ± 92,635	4,176,313 ± 63,599	2716	2734	fatty, soapy, coconut	RI, MS, Tent
**Heterocyclics**						
2-Methylfuran	286,945 ± 28,436	1,597,938 ± 83,305	848	875	chocolate	RI, MS, Tent
2,5-Dimethyl-furan	195,451 ± 8144	462,775 ± 62,104	934	958	chemical, meaty, gravy	RI, MS, STD
2-Vinylfuran	-	3,956,245 ± 32,229	1059	1085	coffee	RI, MS, STD
2-Pentylfuran	30,803,201 ± 11,172	17,918,860 ± 771,626	1209	1241	green bean, butter	RI, MS, STD
Furfural	1,337,092 ± 48,496	16,802,768 ± 25,588	1459	1486	almond	RI, MS, STD
2-Acetyl-5-methylfuran	88,602 ± 8055	402,960 ± 26,362	1612	1608	musty, nutty, coconut	RI, MS, STD
2-Furanmethanol	96,955 ± 4040	419,643 ± 56,316	1645	1666	burnt	RI, MS, STD
1-(5-Methyl-2-furanyl)-propanone	-	1,748,396 ± 14,243	1671	1686	green, hazelnut-like	RI, MS, Tent
2-Pentanoylfuran	176,476 ± 7353	106,465 ± 14,288	1750	1747	sweet caramel	RI, MS, Tent
2 (5H)-Furanone	106,282 ± 4429	234,854 ± 31,517	1761	1787	buttery	RI, MS, STD
2-Hexanoyl furan	312,315 ± 30,950	183,949 ± 9590	1858	1872	sweet, fruity, green	RI, MS, Tent
2-Acetylpyrrole	1,648,279 ± 59,782	137,960 ± 2101	1987	2002	popcorn-like	RI, MS, STD
**Sulfur compounds**						
Methanethiol	283,822 ± 28,127	289,490 ± 15,092	602	643	sulfur, gasoline, garlic	RI, MS, STD
Dimethyl disulfide	316,357 ± 31,351	891,894 ± 46,497	1059	1078	onion, cabbage, putrid	RI, MS, STD
Dimethyl trisulfide	338,026 ± 33,498	763,285 ± 39,792	1353	1400	sulfur, fish, cabbage	RI, MS, STD
2-Pentyl-thiophene	-	667,400 ± 54,360	1422	1460	sweet, fruity	RI, MS, Tent
**Phenols**						
4-Ethyl-2-methoxy-phenol	-	318,607 ± 25,955	2019	2048	smoky, clove	RI, MS, STD
2-Methoxy-4-vinylphenol	7,974,386 ± 28,922	647,661 ± 27,890	2214	2213	smoky, clove	RI, MS, STD
Vanillin	127,696 ± 5321	736,429 ± 98,828	2551	2530	sweet	RI, MS, STD
**Pyrazines**						
2,6-Dimethyl-pyrazine	-	4,684,846 ± 38,164	1335	1308	roasted nut, cocoa	RI, MS, STD
Tetramethyl-pyrazine	59,882 ± 5444	1,142,923 ± 74,771	1472	1462	nutty, coffee, burnt	RI, MS, STD
**Lactones**						
γ-Octalactone	3,815,443 ± 13,838	284,300 ± 4330	1941	1924	coconut milk	RI, MS, STD
γ-Nonalactone	7,974,386 ± 28,922	647,661 ± 27,890	2053	2044	coconut milk	RI, MS, STD

^a^ Cal RI: calculated linear retention indices. ^b^ Lit RI: literature linear retention indices obtained from the NIST library (https://webbook.nist.gov/chemistry/, (accessed on 7 Auguest 2021)). ^c^ The aroma characteristic of the compound itself. ^d^ Identification: tentative identification (Tent.) based on retention indices (RI) and mass spectra (MS), positive identification based on retention times of authentic standards (STD).

**Table 3:** Aroma compounds in cooked glutinous and non-glutinous sorghum.

No. ^a^	Compound	Aroma Descriptor	Aroma Intensity ^b^		Significant ^c^	LRI ^d^	Identification
			Glutinous Sorghum	Non-Glutinous Sorghum			
**Aldehydes**							
1	2-Methylbutanal	Green, malty	3.25 ± 0.27	3.08 ± 0.20	ns	912	MS, RI, Aroma, STD
2	3-Methylbutanal	Green, malty	4.08 ± 0.49	4.17 ± 0.25	ns	925	MS, RI, Aroma, STD
4	Hexanal	Grassy, green	2.92 ± 0.38	2.58 ± 0.37	ns	1084	MS, RI, Aroma, STD
11	Octanal	Fatty, soapy, orange	2.83 ± 0.41	3.17 ± 0.25	ns	1289	MS, RI, Aroma, STD
13	(*E*)-2-Heptenal	Fatty	2.83 ± 0.61	2.00 ± 0.45	*	1315	MS, RI, Aroma, STD
15	Nonanal	Green	4.33 ± 0.52	2.50 ± 0.20	***	1396	MS, RI, Aroma, STD
17	(*E*)-2-octenal	Green	4.58 ± 0.38	4.00 ± 0.45	*	1435	MS, RI, Aroma, STD
22	Decanal	Fatty/waxy	2.50 ± 0.45	2.08 ± 0.51	ns	1506	MS, RI, Aroma, STD
23	Benzaldehyde	Almond	5.00 ± 0.00	4.33 ± 0.25	**	1541	MS, RI, Aroma, STD
30	(*E*)-2-decenal	Green	1.00 ± 0.71	0.83 ± 0.45	ns	1656	MS, RI, Aroma, STD
31	Phenylacetaldehyde	Rose	1.50 ± 0.45	2.00 ± 0.38	ns	1675	MS, RI, Aroma, STD
33	(*E*, *E*)-2,4-Nonadienal	Green, fatty	2.83 ± 0.61	3.08 ± 0.45	ns	1711	MS, RI, Aroma, STD
37	(*E*)-2-Undecenal	Soapy, metallic	-	0.92 ± 0.32	***	1736	MS, RI, Aroma, STD
38	(*E*, *E*)-2,4-Decadienal	Fatty, vegetable	1.75 ± 0.27	-	***	1820	MS, RI, Aroma, STD
**Terpenes**							
5	α-Terpinene	Citrus-like, floral	1.83 ± 0.61	1.50 ± 0.38	ns	1185	MS, RI, Aroma, STD
6	Terpinolene	Citrus-like, floral	1.17 ± 0.61	1.00 ± 0.32	ns	1200	MS, RI, Aroma, STD
7	α-Limonene	Citrus-like	1.25 ± 0.69	-	**	1270	MS, RI, Aroma, STD
9	γ-Terpinene	Citrus-like, lemon	2.25 ± 0.52	2.00 ± 0.41	ns	1273	MS, RI, Aroma, STD
24	(-)-α-Cedrene	Cypress	5.00 ± 0.00	3.17 ± 0.40	***	1574	MS, RI, Aroma, STD
25	Thujopsene	Cypress	1.50 ± 0.45	-	***	1620	MS, RI, Aroma, STD
28	β-Terpineol	Clove	0.92 ± 0.49	1.33 ± 0.38	ns	1626	MS, RI, Aroma, STD
36	β-Elemene	Fruity, sweet	1.83 ± 0.41	-	***	1598	MS, RI, Aroma, STD
39	β-Damascenone	Sweet, honey	5.00 ± 0.00	4.08 ± 0.45	**	1833	MS, RI, Aroma, STD
42	(*E*)-β-Ionone	Violet	2.25 ± 0.52	1.75 ± 0.38	ns	1952	MS, RI, Aroma, STD
45	Geranyl acetone	Fruity	5.00 ± 0.00	4.25 ± 0.25	***	1856	MS, RI, Aroma, STD
46	(*E*)-Nerolidol	Rose, apple	1.50 ± 0.45	1.92 ± 0.37	ns	2015	MS, RI, Aroma, STD
52	Vanillin	Sweet	1.83 ± 0.61	2.17 ± 0.51	ns	2542	MS, RI, Aroma, STD
**Acids**							
20	Acetic acid	Acid	0.83 ± 0.52	1.50 ± 0.49	ns	1455	MS, RI, Aroma, STD
29	Butanoic acid	Sweaty, acid	2.17 ± 0.41	2.67 ± 0.66	ns	1657	MS, RI, Aroma, STD
34	3-Methyl-butanoic acid	Acid	2.25 ± 0.52	2.50 ± 0.55	ns	1687	MS, RI, Aroma, STD
35	Pentanoic acid	Sweaty	2.25 ± 0.27	2.58 ± 0.45	ns	1754	MS, RI, Aroma, STD
40	Hexanoic acid	Sweaty, cheese	5.00 ± 0.00	5.00 ± 0.00	ns	1844	MS, RI, Aroma, STD
43	Heptanoic acid	Sweaty	1.67 ± 0.26	2.17 ± 0.68	ns	1915	MS, RI, Aroma, STD
49	Octanoic acid	Grease	1.75 ± 0.61	2.08 ± 0.68	ns	2060	MS, RI, Aroma, STD
51	Decanoic acid	Grease	3.17 ± 0.68	3.50 ± 0.45	ns	2284	MS, RI, Aroma, STD
**Ketones**							
10	2-Octanone	Milk	1.58 ± 0.49	1.83 ± 0.58	ns	1282	MS, RI, Aroma, STD
12	2, 3-Octanedione	Cream, sweet	3.33 ± 0.41	2.67 ± 0.20	**	1368	MS, RI, Aroma, STD
14	2-Nonanone	Sweet, coconut	1.17 ± 0.41	1.00 ± 0.45	ns	1388	MS, RI, Aroma, STD
27	2-Undecanone	Orange	1.17 ± 0.61	1.58 ± 0.45	ns	1596	MS, RI, Aroma, STD
32	Acetophenone	Floral	4.00 ± 0.45	3.08 ± 0.51	*	1668	MS, RI, Aroma, STD
**Alcohols**							
16	Hexanol	Floral	3.50 ± 0.71	3.00 ± 0.38	ns	1376	MS, RI, Aroma, STD
18	1-Octen-3-ol	Mushroom	3.33 ± 0.52	3.50 ± 0.38	ns	1440	MS, RI, Aroma, STD
19	Heptanol	Green	0.92 ± 0.49	1.17 ± 0.38	ns	1450	MS, RI, Aroma, STD
26	Octanol	Fruity	2.50 ± 0.63	2.75 ± 0.51	ns	1575	MS, RI, Aroma, STD
41	Phenethyl alcohol	Honey	3.50 ± 0.45	4.17 ± 0.60	ns	1942	MS, RI, Aroma, STD
**Heterocyclics**							
8	2-Pentylfuran	Grass, bean	2.92 ± 0.58	2.00 ± 0.38	*	1218	MS, RI, Aroma, STD
21	Furfural	Almond	1.17 ± 0.61	1.00 ± 0.58	ns	1464	MS, RI, Aroma, STD
44	2-Acetylpyrrole	Popcorn-like	0.75 ± 0.42	1.83 ± 0.45	**	1957	MS, RI, Aroma, STD
47	γ-Octalactone	Coconut milk	3.50 ± 0.63	3.00 ± 0.74	ns	1886	MS, RI, Aroma, STD
48	γ-Nonalactone	Coconut milk	2.92 ± 0.58	2.50 ± 0.38	ns	2059	MS, RI, Aroma, STD
**Sulphur**							
3	Dimethyl disulfide	Onion	2.00 ± 0.32	1.17 ± 0.38	**	1078	MS, RI, Aroma, STD
**Aromatic**							
50	2-Methoxy-4-vinylphenol	Smoky, clove-like	2.25 ± 0.52	2.50 ± 0.63	ns	2200	MS, RI, Aroma, STD

^a^ Numbers represent the aroma compounds listed in Figure 4; ^b^ The aroma intensities of odorant compounds were represented as the mean value of sextuplicate samples ± standard deviation; ^c^ Symbols indicates a significant aroma intensity difference between two sorghum samples. Significance: “***”, *p* < 0.001 (very highly significant), “**”, *p* < 0.01 (highly significant) “*”, *p* < 0.05 (significant); “ns”, *p* > 0.05 (not significant). ^d^ LRI: linear retention indices.

**Table 4:** Summed aroma intensities of each aroma group present in the cooked glutinous and non-glutinous sorghum samples.

Aroma Impression ^a^	Volatile Compounds	Summed Aroma Intensities ^b^		Significance ^c^
		Glutinous	Non-Glutinous	
sweet/fruity	2-octanone, 2,3-octanedione, 2-nonanone, octanol, β-elemene, β-damascenone, phenethyl alcohol, geranyl acetone, γ-octalactone, γ-nonalactone, vanillin	32.17 ± 2.32	28.42 ± 1.48	*
green	2-methylbutanal, 3-methylbutanal, hexanal, 2-pentylfuran, nonanal, (*E*)-2-octenal, 1-heptanol, (-)-α-cedrene, thujopsene, (*E*)-2-decenal, (*E*, *E*)-2,4-nonadienal	33.33 ± 1.75	26.58 ± 1.51	***
floral	hexanol, β-terpineol, phenylacetaldehyde, acetophenone, (E)-β-ionone, (*E*)-nerolidol	13.67 ± 1.67	13.08 ± 1.51	ns
fatty	octanal, (*E*)-2-heptenal, decanal, (E)-2-undecenal, (*E*, *E*)-2,4-decadienal, octanoic acid, decanoic acid	14.83 ± 0.75	13.75 ± 1.80	ns
volatile acids	acetic acid, butanoic acid, 3-methyl-butanoic acid, pentanoic acid, hexanoic acid, heptanoic acid	14.17 ± 0.75	16.42 ± 1.06	**
citrus-like	α-terpinene, (*E*)-β-terpinolene, α-limonene, γ-terpinene, 2-undecanone	7.67 ± 0.55	6.08 ± 0.53	***
roasty/nutty	furfural, benzaldehyde, 2-acetylpyrrole	6.92 ± 0.61	7.17 ± 0.69	ns
miscellaneous (sulphury/smoky/mushroom)	dimethyl disulfide, 1-octen-3-ol, 2-methoxy-4-vinylphenol	7.58 ± 0.79	7.17 ± 0.47	ns

^a^ The aroma characteristics; ^b^ The summed aroma intensities of odorant compounds were represented as the mean value of sextuplicate samples ± standard deviation; ^c^ Symbols indicate a significant aroma intensity difference between two sorghum samples. Significance: “***”, *p* < 0.001 (very highly significant); “**”, *p* < 0.01 (highly significant) “*”, *p* < 0.05 (significant); “ns”, *p* > 0.05 (not significant).

## Data Availability

The data presented in this study are available on request from the corresponding author.

## Sample Availability

Samples of the compounds are not available from the authors.
